# Emodin-8-*O*-Glucoside—Isolation and the Screening of the Anticancer Potential against the Nervous System Tumors

**DOI:** 10.3390/molecules28217366

**Published:** 2023-10-31

**Authors:** Estera Okon, Maryna Koval, Anna Wawruszak, Adrianna Slawinska-Brych, Katarzyna Smolinska, Myroslav Shevera, Andrzej Stepulak, Wirginia Kukula-Koch

**Affiliations:** 1 Department of Biochemistry and Molecular Biology, Medical University of Lublin, 20-093 Lublin, Poland; estera.okon@umlub.pl (E.O.); anna.wawruszak@umlub.pl (A.W.); 2 Department of Pharmacognosy with Medicinal Plants Garden, Medical University of Lublin, 20-093 Lublin, Poland; marinchik.koval@gmail.com; 3 Department of Cell Biology, Maria Curie-Sklodowska University, 20-031 Lublin, Poland; adrianna.slawinska-brych@mail.umcs.pl; 4 Chronic Wounds Laboratory, Medical University of Lublin, 20-093 Lublin, Poland; katarzyna.smolinska@umlub.pl; 5 M.G. Kholodny Institute of Botany of the National Academy of Sciences of Ukraine, 2, Tereshchenkivska Str., 010601 Kyiv, Ukraine; shevera.myroslav@ukr.net

**Keywords:** emodin-8-*O*-glucoside, Polygonaceae, anthraquinones, centrifugal partition chromatography, anticancer activity, nervous system tumors

## Abstract

Emodin-8-*O*-glucoside (E-8-*O*-G) is a glycosylated derivative of emodin that exhibits numerous biological activities, including immunomodulatory, anti-inflammatory, antioxidant, hepatoprotective, or anticancer activities. However, there are no reports on the activity of E-8-*O*-G against cancers of the nervous system. Therefore, the aim of the study was to investigate the antiproliferative and cytotoxic effect of E-8-*O*-G in the SK-N-AS neuroblastoma, T98G human glioblastoma, and C6 mouse glioblastoma cancer cells. As a source of E-8-*O*-G the methanolic extract from the aerial parts of *Reynoutria japonica* Houtt. (Polygonaceae) was used. Thanks to the application of centrifugal partition chromatography (CPC) operated in the descending mode using a mixture of petroleum ether:ethyl acetate:methanol:water (4:5:4:5 *v*/*v*/*v*/*v*) and a subsequent purification with preparative HPLC, E-8-*O*-G was obtained in high purity in a sufficient quantity for the bioactivity tests. Assessment of the cancer cell viability and proliferation were performed with the MTT (3-(bromide 4,5-dimethylthiazol-2-yl)-2,5-diphenyltetrazolium), CTG (CellTiter-Glo^®^) and BrdU (5-bromo-2′-deoxyuridine) assays, respectively. E-8-*O*-G inhibits the viability and proliferation of SK-N-AS neuroblastoma, T98G human glioblastoma multiforme, and C6 mouse glioblastoma cells dose-dependently. E-8-*O*-G seems to be a promising natural antitumor compound in the therapy of nervous system tumors.

## 1. Introduction

Plants are rich sources of numerous and diverse chemical compounds which show plenty of biological activities. The search for new drug candidates that could help in the treatment of civilization diseases is of utmost importance these days [1]. Among the metabolites of plant origin that have been found efficient in the treatment of cancer polyphenols [2], alkaloids [3], saponins [4], and anthracene derivatives [5] were listed. The latter were identified in various representatives of the Polygonaceae, Rubiaceae, Rhamnaceae, Fabaceae, Liliaceae, or Scrophulariaceae botanical families [6].

Anthraquinones are an important class of natural and semi-synthetic with a wide range of applications [6]. They are characterized by high structural diversity and promising therapeutic potential. Several anthraquinone derivatives, such as daunomycin, doxorubicin, or mitoxantrone, are widely used as chemotherapeutic agents [7].

Emodin is an anthraquinone derivative isolated from the roots and bark of, e.g., *Rheum palmatum*, Aloe vera, Giant knotweed, *Polygonum multiflorum*, *Polygonum cuspidatum*, etc. [8]. It possesses a wide range of biological activities including antiviral [9], antibacterial [10], anti-inflammatory [11], or anticancer properties [12]. It has been demonstrated that emodin was active against liver [13], lung [14], breast [15], and ovarian cancer cells [16]. Its role in chemotherapy has been explored also in synergism with various allopathic drugs. The use of emodin in the above-mentioned therapies minimized their toxicity and increased their effectiveness [12].

**Figure 1:**
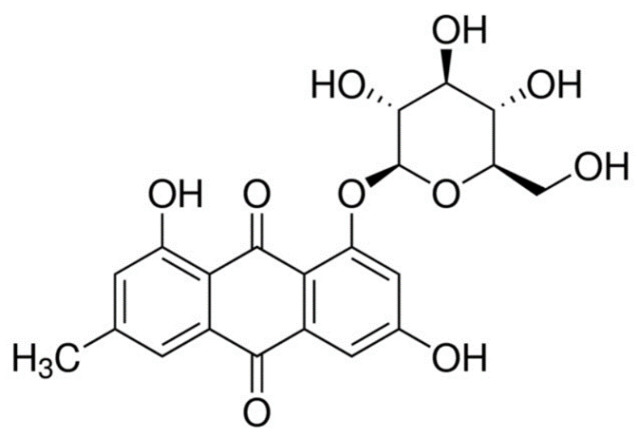
The structure of emodin-8-*O*-glucoside (E-8-*O*-G).

Unfortunately, in vitro and in vivo toxicity analyses showed that emodin when used for a long time can adversely affect the reproductive system, kidneys, and liver. Therefore, its glycosylated derivatives are tested to reduce its toxicity. It has been demonstrated that glycosylation of anthraquinones affects their biological, physical, and chemical functions [17]. Glycosylation of emodin, which is one of the most studied aglycones that belong to this group, by the plants can lead to the formation of glycosylated derivatives, for example emodine glucosides, like emodin-8-*O*-glucoside (E-8-*O*-G) (Figure 1) or emodin-6-*O*-glucoside [17].

Existing literature data about the effects of anthracene derivatives on the central nervous system functions confirm their ability to penetrate the blood–brain barrier (BBB) after a parenteral administration. It has been shown that E-8-*O*-G may deliver a neuroprotective effect against cerebral ischemia-reperfused injury and glutamate-induced neuronal damage in vivo [18]. These data encouraged the authors to study the potential of E-8-*O*-G in the treatment of nervous system-related cancers.

This direction of research was also encouraged by other scientific reports confirming the immunomodulatory properties of E-8-*O*-G in the mouse RAW264.7 macrophages and THP-1 human monocytic cells. The results proved that E-8-*O*-G (20 μM) induced tumor necrosis factor α (TNF-α) and interleukin-6 (IL-6) secretion in RAW264.7 cells. Additionally, E-8-*O*-G significantly increased Toll-like receptor 2 (TLR-2) mRNA expression and mitogen-activated protein kinases (MAPK) phosphorylation (c-jun N-terminal kinase (JNK) and p38). All these results suggest that E-8-*O*-G may represent a novel immunomodulator enhancing early innate immunity [17]. However, there are no reports on the activity of E-8-*O*-G against cancers of the nervous system.

As a source of E-8-*O*-G, *Reynoutria japonica* Houtt. (syn: *Polygonum cuspidatum*, *Fallopia japonica*, *Polygonum reynoutria*, etc.) (Polygonaceae) was selected. This herbaceous perennial plant is considered a dangerous and invasive species in many countries of the world. The plant is native to East Asia in Japan, China, and Korea. For a long time, it has been used in traditional medicine in China, where it is known as *Huzhang*. *Polygonum cuspidatum* exhibits many pharmacological effects, like the anti-inflammatory, antioxidant, hepatoprotective, lipid regulating, anti-shock activity, antiviral, antibacterial, antifungal, and anticancer properties that could be induced by the presence of a wide range of secondary metabolites from the groups of flavonoids, coumarins, lignans, stilbenes, quinones, and others [19]. The pharmacognostic portfolio of the plant and the fact that is perceived as an invasive plant encouraged the authors to make use of its abundantly growing overground parts and to use them for the large-scale recovery of metabolites of pharmacological significance. For this purpose, centrifugal partition chromatography (CPC) was applied, which an easily upscaleable technique and promises easy upscaling from analytical to industrial conditions. This can be achieved by its operational characteristics. CPC uses a biphasic solvent system that plays the role of a stationary and mobile phase and guarantees high sample recovery due to the lack of solid adsorbent. Low-temperature operation, no need for high-purity solvents, and high selectivity of the composed mixtures of solvents make this technique suitable for the recovery of single components directly from crude extracts of plant origin [20].

Therefore, in our studies, we determined an anticancer effect of E-8-*O*-G for the first time, which was possible thanks to the optimization of its recovery protocol from the methanolic extract from the aerial parts of *Reynoutria japonica* by CPC and preparative HPLC techniques. The compound of interest was used for the experimental treatment against SK-N-AS neuroblastoma, T98G human glioblastoma, and C6 mouse glioblastoma cancer cells.

## 2. Results

### 2.1. Composition of the Extract

The parameters of the chromatographic analysis of the methanolic extract from the aerial parts of *Reynoutria japonica* provided a clear separation of the extract’s constituents.

**Figure 2:**
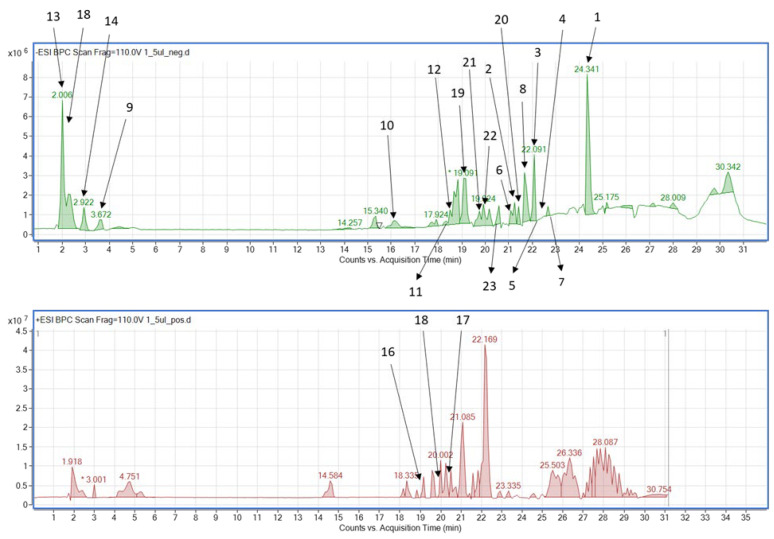
The total ion chromatograms of the tested extract in both negative (**above**) and positive (**below**) ionization modes. (* manually integrated peaks).

The plant was found to be a rich source of polyphenols, including phenolic acids, flavonoids, flavonoid glucosides, anthracene derivatives, and their glucosides, but also coumarins and organic acids. Among the major representatives of the secondary metabolites procyanidins were traced in the positive ionization mode, whereas anthraquinones like emodin and its glucosides and luteolin glucoside were the leading components of the extract (Figure 2 and Table 1).

As previously mentioned by other researchers, the analyzed extract was found to contain similar classes of compounds, as described in a review manuscript of Nawrot-Hadzik and collaborators [21,22].

### 2.2. The Fractionation of the Extract by CPC and Preparative HPLC

The final composition of a separating system was elaborated based on the scientific publications that reported the purification of anthracene derivatives by high performance counter current chromatography, which is a hydrodynamic variation of counter-current chromatography. The suitability of their composition was performed using a shake-flask method on the seven selected systems (see Supplementary Materials Table S2). For this purpose, every mixture of solvents was prepared in the volume of 5 mL in a glass tube and was mixed with ca. 10 mg of the extract. After the dissolution of the sample, the upper and lower phases were separately analyzed by HPLC-MS to observe the affinity of the extract’s constituent to each phase and calculate the partition coefficient values of every major component. The system that was found to separate emodin glucosides from the rest of the extract’s components was selected for the fractionation of the total extract.

**Figure 3:**
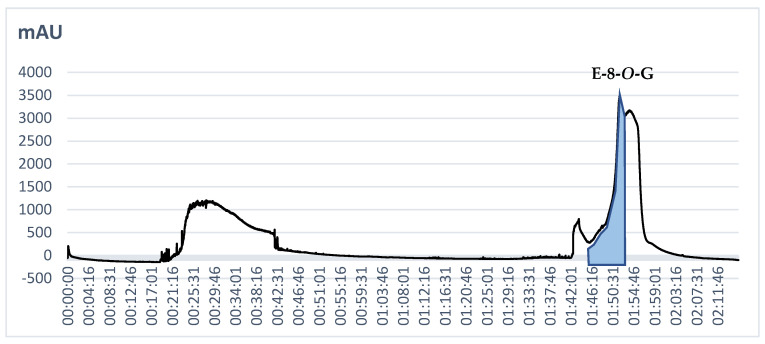
Chromatogram obtained in the CPC separation of the methanolic extract from *Reynoutria japonica* aerial parts at 290 nm.

**Figure 4:**
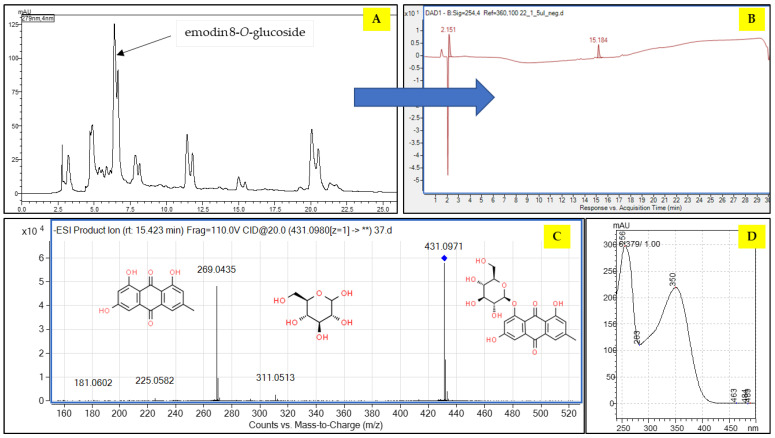
Chromatogram obtained in the preparative HPLC separation of the methanolic extract from *Reynoutria japonica* aerial parts (**A**), the HPLC chromatogram of the isolated emodin 8-*O*-glucoside (**B**), the MS/MS fragmentation pattern of the compound confirming its identity (**C**), and the UV spectrum of the isolated emodin 8-*O*-glucoside (**D**) (** manually integrated peak).

Finally, the solvent system composed of petroleum ether: ethyl acetate: methanol: water (4:5:4:5 *v*/*v*/*v*/*v*) in the descending mode provided fractions that were enriched in emodin glucosides. Two compounds (emodin 6-*O*-glucoside and 8-*O*-glucoside) were obtained from 109 to 113 min after the start of the analysis (Figure 3), and they were clearly visible in three CPC fractions (37–40) (Figure 3 and Figure 4).

The fraction that was enriched in emodin glucosides was evaporated to dryness on a rotary evaporator at 45 °C, resuspended in a mixture of water: methanol 70:30 (*v*/*v*) and subjected to preparative HPLC separation. The second step enabled a precise purification of E-8-*O*-G from the fraction, which is proven in Figure 3. The purity of the isolate was calculated as 96.5%.

### 2.3. Cytotoxic Activity of the E-8-O-G

**Figure 5:**
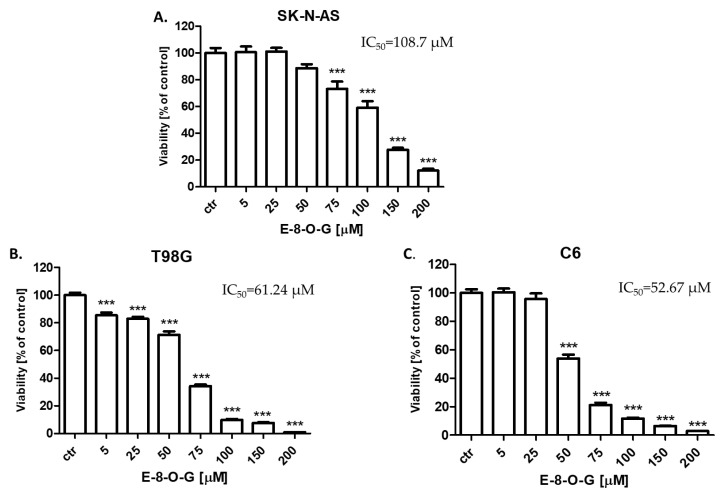
The effect of emodin-8-*O*-glucoside (E-8-*O*-G) (5–200 µM) on the viability of SK-N-AS neuroblastoma (**A**), T98G human glioblastoma (**B**), and C6 mouse glioblastoma (**C**) cells after 96 h incubation in the MTT assay. Data are presented as mean ± standard deviation (±SD); one-way ANOVA, Tukey post hoc testing; *** *p* < 0.001.

The antiproliferative activity of E-8-*O*-G (5–200 µM) for 96 h was determined in SK-N-AS neuroblastoma, T98G human glioblastoma and C6 mouse glioblastoma cancer cells using the MTT assay to establish the IC_50_ value for E-8-*O*-G (Figure 5). E-8-*O*-G (5–200 µM) inhibited in a dose-dependent manner the viability of all analyzed neoplastic cells of the nervous system in the MTT test. The highest activity of E-8-*O*-G was observed against C6 mouse glioblastoma cell line with IC_50_ = 52.67 µM (Figure 5C). The IC_50_ for T98G human glioblastoma cells and SK-N-AS neuroblastoma cells in the MTT assay were 61.24 µM (Figure 5B) and 108.7 µM (Figure 5A), respectively.

**Figure 6:**
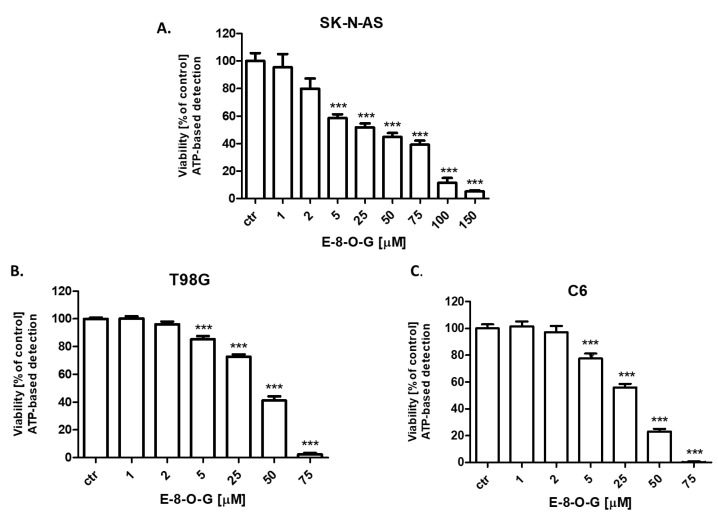
The effect of emodin-8-*O*-glucoside (E-8-*O*-G) (1–150 µM) on the viability of SK-N-AS neuroblastoma (**A**), T98G human glioblastoma (**B**) and C6 mouse glioblastoma (**C**) cells after 96 h incubation in the CellTiter-Glo^®^ assay. Data are presented as mean ± standard deviation (±SD); one-way ANOVA, Tukey post hoc testing; *** *p* < 0.001.

The cytotoxic activity of E-8-*O*-G was confirmed in the CellTiter-Glo^®^ assay. E-8-*O*-G in doses of 1–150 µM inhibited dose-dependently the viability of SK-N-AS neuroblastoma (Figure 6A), T98G human glioblastoma (Figure 6B), and C6 mouse glioblastoma (Figure 6C) cells, inhibiting the production of ATP in all analyzed cancer cells. E-8-*O*-G showed the highest activity against SK-N-AS neuroblastoma cells (Figure 6A), followed by C6 mouse glioblastoma (Figure 6C) and T98G human glioblastoma multiforme (Figure 6B) cells.

### 2.4. Antiproliferative Activity of the E-8-O-G

**Figure 7:**
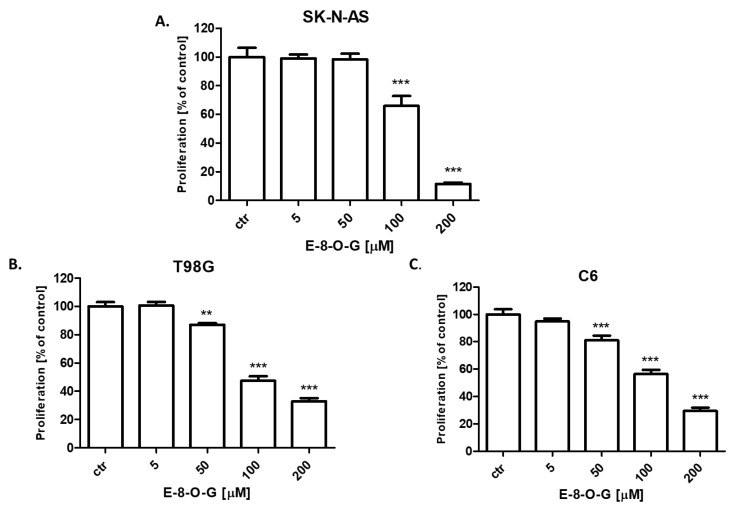
The effect of emodin-8-*O*-glucoside (E-8-*O*-G) (5–200 µM) on the proliferation of SK-N-AS neuroblastoma (**A**), T98G human glioblastoma (**B**) and C6 mouse glioblastoma cells (**C**) after 96 h incubation in the BrdU assay. Data are presented as mean ± standard deviation (±SD); one-way ANOVA, Tukey post hoc testing; ** *p* < 0.01, *** *p* < 0.001.

The effect of E-8-*O*-G on cancer cell proliferation was attributed to decreased cell division, as determined by the decreased incorporation of BrdU. SK-N-AS neuroblastoma, T98G human glioblastoma, and C6 mouse glioblastoma cancer cells were exposed to either culture medium (control) or E-8-*O*-G in the concentration range from 5 to 200 µM. E-8-*O*-G reduced the proliferation of all analyzed cancer cells in a dose-dependent manner after 48 h of incubation. In the case of the C6 (Figure 7C) and T98G (Figure 7B) cell lines, statistical significance was observed at concentrations of 50, 100, and 200 µM. In turn, in the SK-KN-AS (Figure 7A) cell line, only in the case of concentrations of 100 and 200 µM statistical significance was noticed.

## 3. Discussion

Insufficient effectiveness of commonly used anticancer therapies or their negative side effects have resulted in a great interest in natural medicine. Modern pharmacognosy and phytotherapy are looking for new bioactive substances of plant origin with promising chemotherapeutic parameters [1].

Among the compounds of plant origin, the secondary metabolites that are produced by plants under stress conditions are potential therapeutic molecules that can be applied in the treatment of various human diseases [23]. Also, anthracene derivatives have been discovered to play an important role in the treatment of cancer [6]. These compounds are present in the form of aglycones or glucosides in the plant material. Depending on their form and their hydrogenation level, these compounds can be characterized by different solubility or exhibited pharmacological action. For the moment, various representatives of several botanical families were proven to contain anthracene derivatives, like anthraquinones or anthrons. Among them, *Rubiaceae*, *Fabaceae*, *Rhamnaceae*, *Polygonaceae*, *Liliaceae*, or others contain species that the most known sources of anthracene derivatives [6]. *Reunoutria japonica*—a representative of *Polygonaceae*—became a species of interest for the purification of emodin glucosides as shown above. The plant originates from Asia; however, from the 19th century, it has been widely spread in temperate climates of Europe as well. Proved to be an invasive species, *R. japonica* is a ruderal plant is characterized by a rich fingerprint of polyphenols. As described above and by other authors, the plant contains anthracene derivatives, and among them E-8-*O*-G, a glycosylated derivative of emodin. Currently, most research focuses on the activity of emodin itself, while there are still limited reports on the activity of its glycosylated form [18,24].

The lack of the studies on this molecule is caused by a small quantity of the compound in the total extract. This was also the case of *R. japonica* samples investigated in our study. Rich composition in secondary metabolites of similar chemical character makes it difficult to isolate sufficient quantities of E-8-*O*-G from the total extract.

CPC enabled the realization of this task. The technique is a solid support liquid-liquid fractionation technique where the separation of mixture constituents takes place on a rotating column in a biphasic solvent system with one immobilized solvent layer inside a rotating column and another one flushing the stationary one. According to the affinity of the metabolites to the upper or lower phase, the separation occurs. It is important to underline that CPC is easily up-scaleable and operates using reagent-grade solvents, which are of low cost. In such conditions, the injection of a high concentration of crude extract may deliver large quantities of enriched fractions that can be later purified using, e.g., preparative HPLC technique.

Previously, other authors reported the isolation of emodin from different plant species using a hydrodynamic liquid–liquid separation technique, namely HSCCC. The system composed of hexane: ethyl acetate: methanol: water (3:7:5:5, 9:1:5:5, *v*/*v*/*v*/*v*) was found efficient for the fractionation of emodin from the extract of *Polygonum multiflorum* in a stepwise elusion [25]. The other authors were successful in operating an HSCCC apparatus in the isolation of anthraquinones from *Polygonum cuspidatum*. By using the biphasic solvent system composed of ethyl acetate:methanol:water (70:1:70, *v*/*v*/*v*/*v*) they obtained high purity E-8-*O*-G and partially purified sample, from which managed to separate emodin-6-*O*-glucoside with help of petroleum ether:ethyl acetate:water (1:5:5, *v*/*v*/*v*/*v*) from a single injection of crude plant extract [26].

In our case, the application of a solvent system composed of petroleum ether:ethyl acetate:methanol:water (4:5:4:5 *v*/*v*/*v*/*v*) that was previously used by Minzhuo Liu et al. [27] for the fractionation of *Polygonum multiflorum* metabolites succeeded in the recovery of a fraction enriched in E-8-*O*-G from *Reynoutria japonica.* The fraction could be then purified by preparative HPLC to obtain a high-purity metabolite in the quantity sufficient for in vitro tests.

In our studies, the anticancer activity of E-8-*O*-G isolated from *Reynoutria japonica* using hyphenated chromatographic techniques against the SK-N-AS neuroblastoma, T98G human glioblastoma, and C6 mouse glioblastoma was determined. E-8-*O*-G inhibited the viability and proliferation of all tested cancer cells dose-dependently in MTT, CellTiter-Glo^®^, and BrdU assays, respectively. In the MTT test, E-8-*O*-G showed the highest activity against the C6 mouse glioblastoma cell line, where the IC_50_ (half maximal inhibitory concentration) was the lowest (52.67 µM). The IC_50_ for T98G human glioblastoma cells and SK-N-AS neuroblastoma cells were 61.24 µM and 108.7 µM, respectively. Similarly to our results, E-8-*O*-G was reported to have antitumor activity against human ovarian cancer. It has been demonstrated that E-8-*O*-G inhibited the proliferation and promoted apoptosis in SKOV3 cells. Additionally, E-8-*O*-G caused down-regulation of Bcl-2 (B-cell lymphoma 2) expression and up-regulation of Bax protein expression, as well as the increase in relative activity of caspase 3 and 9 [28].

It has been demonstrated that E-8-*O*-G was able to provide neuroprotection against cerebral ischemia-reperfused injury and glutamate-induced neuronal damage by exerting antioxidative effects and inhibiting glutamate neurotoxicity. The results of this study revealed that the treatment of rats with E-8-*O*-G reduced the cerebral infarction area and the neurological deficit score, as well as increased superoxide dismutase activity and total antioxidative capability, and decreased malondialdehyde level in the brain tissue in a dose-dependent fashion. Moreover, E-8-*O*-G inhibited the neuronal damage induced by glutamate. Importantly, E-8-*O*-G could penetrate the blood–brain barrier (BBB) and distribute in the brain tissue [18].

Additionally, non-glucosylated emodin inhibits U87 glioblastoma cell migration by activating the aryl hydrocarbon receptor (AhR) signaling pathway [29]. Emodin also significantly enhanced the radiosensitivity of LN18 and LN428 glioblastoma cells to γ-rays. Exposure of cancer cells to neutron radiation in the presence of emodin induced autophagic and apoptotic cell death, and suppressed cell migration and invasiveness [30].

Exposure of U87MG glioblastoma cells to another emodin derivative, aloe-emodin, significantly decreased the proliferation of U87MG cells time- and dose-dependently, blocked off the cell cycle in the S and G2/M phase, and induced apoptosis [31]. Moreover, the additive inhibitory effect of aloe-emodin and temozolomide was confirmed in the NULU and ZAR patient-derived glioma cell lines, which exhibit drug-resistant phenotypes. Furthermore, scratch tests and migration assays showed that the combined use of these agents can slow down the colony formation and migration of glioblastoma cells [32].

All these findings indicate that E-8-*O*-G may be a promising therapeutic adjuvant for the treatment of certain cancers of the nervous system. However, the mechanism of action of E-8-*O*-G, alone or in combination with other active substances, should be verified in in vivo and in vitro functional studies.

## 4. Materials and Methods

### 4.1. Reagents

All reagents used for the extraction and CPC fractionation were of analytical grade (Avantor Performance Materials, Gliwice, Poland). Water used for chromatography experiments was redistilled. Methanol and acetonitrile for preparative HPLC were of HPLC grade, whereas acetonitrile and water for LC-MS analyses were of LC-MS grade. They were all purchased from Merck (Darmstadt, Germany).

### 4.2. Plant Material and Extraction of Reynoutria japonica Herb

The plant material was obtained from the Department of Systematics and Floristics of Vascular Plants of M.H. Kholodny Institute of Botany, National Academy of Sciences of Ukraine in Kyiv, where it was authenticated by Myroslav Shevera. The overground parts of *R. japonica* were collected in Ukraine, Transcarpathian Region, Uzhhorod, near park “Bozdosh” in ruderal places in September 2022 by M. Shevera.

The plant material was dried at 30 °C in the air and finely cut, ground and subjected to further studies. Fifty grams of finely crushed plant material was placed in Erlenmeyer flasks, and poured methanol in the ratio of solvent to solute: 10 to 1. The containers were shaken on a laboratory shaker for about 15 min and subjected to ultrasound extraction at room temperature for the following 30 min. The extract was filtered off and the extraction procedure was repeated 3 times. The extracts were collected together and evaporated to dryness on a rotary evaporator at 45 °C.

### 4.3. Fingerprinting of the Extract by HPLC-ESI-QTOF-MS/MS

The extract and all fractions were analyzed by a high-resolution HPLC-MS instrument produced by Agilent Technologies (Santa Clara, CA, USA). The chromatograph was composed of a degasser G1379B, a two-channel pump G1312A, an autosampler G1329A, a thermostate G1316B and a mass detector QTOF with electrospray ionisation G6530B. The analysis was carried out with Zorbax Eclipse Plus RP-18 chromatographic column (150 mm × 2.1 mm, 3.5 µm) by Agilent Technologies. The solvent composition included channel A—water with 0.1% formic acid, channel B—acetonitrile with 0.1% formic acid. The following gradient elution for solvent B was applied: 5 min—20%, 10 min—40%, 17–23.90 min—95%, 24–25 min—1%. The flow rate was 0.2 mL/min, the wavelength detection was set at 254 nm. The details of mass spectrometer settings were: the capillary voltage of 3000 V, the gas and sheath gas temperatures of 275 and 325 °C, the gas flows of 12 L/min, the nebulizer pressure of 35 psig, the skimmer voltage of 65 V, the collision energies (CID) of 10 and 20 V, and the fragmentor voltage of 110 V. The data analysis was performed using a dedicated program Mass Hunter Workstation (version B.10.00) by Agilent Technologies. The identification of the compounds was based on the high resolution mass measurements, the MS/MS fragmentation patterns (presented in the Supplementary Materials Table S1), and in consideration of the previously published compositional data on this particular plant species and the representatives of the same genus.

### 4.4. Fractionation of the Extract by Centrifugal Partition Chromatography (CPC)

The fractionation of the extract was possible thanks to the determination of the final composition of the biphasic solvent system that was pumped on a column. Before, from several compositions of solvents, seven systems were selected to be tested in tubes for their separation efficiency, based on the polarity of the compounds of interest (Table 2).

**Figure 8:**
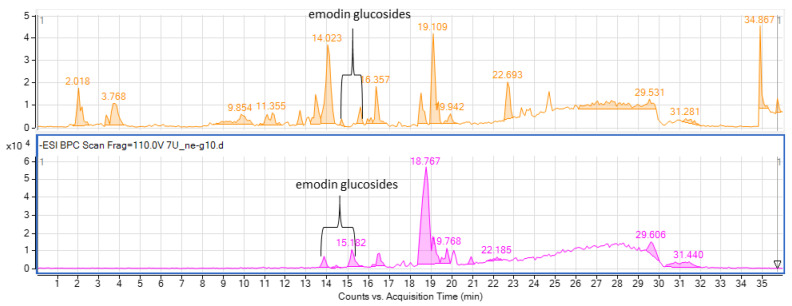
LC-MS chromatogram of the upper and lower phases of the solvent system 7—petroleum ether:ethyl acetate:methanol:water (4:5:4:5 *v*/*v*/*v*/*v*).

The selection systems for CPC were based on a review of the literature and the information on the construction of biphasic solvent systems in the recovery of anthracene derivatives. The above solvent systems were prepared at the volume of 5 mL in test tubes and the dried residue of extract (ca. 20 mg) was diluted in every system. Later, the upper and lower phases were filtered through a nylon syringe filter (pore diameter of 0.1 µm) to an HPLC vial and they were injected into the HPLC-MS to study the efficiency of the separation of metabolites between the upper and lower phases that were expressed by the calculation of partition coefficient value (k) for every major peak visible in the chromatograms (k = peak area of a given peak in the upper phase/peak area of a given peak in the lower phase). Thanks to the best location of emodin glucosides, solvent system no. 7 was selected for further evaluation. The results of HPLC analysis of the selected biphasic solvent systems is presented in Table S2 in the Supplementary File. As shown in Figure 8, emodin glucosides were present in the upper phase in higher concentration, in contrast to the remaining metabolites present in the extract.

Based on the obtained chromatograms, the upper phase that was rich in emodin glucosides was selected as the stationary phase. The CPC separation was carried out with the help of a CPC chromatograph (SCPC-250-L, Armen Instruments, Saint Ave, France), equipped with a 250 mL stainless steel column, a UV detector, and a fraction collector. First, the solvents from the selected system were mixed in a separation funnel. After a complete separation of organic and aqueous layers, they were poured into individual bottles and indicated as upper and lower phases consequently. Later, 2 g of dried crude extract was dissolved in 10 mL of solvents—in equal volumes of the upper and lower phases. Within the first 15 min, the column was filled with a lower stationary phase with a flow rate of 20 mL/min. At the same time, the chromatograph rotor was rotating at 500 rpm. When the column was filled, the mobile phase was pumped for 15 min, and when the hydrodynamic equilibrium was established, the sample was injected into the valve. The separation was conducted at the flowrate rotation of 1000 rpm, where the mobile phase was submitted into the column at a speed of 5 mL/min. The fractions were collected every 5 min using a fraction collector into 32 mL-volumed test tubes. After half an hour of isolation, the mode was changed to extrusion with a flow rate of 10 mL/min. The total analysis lasted 2 h and 15 min (135 min). The wavelength of the detector was set at 254 nm and 290 nm. There were 52 fractions obtained in the experiment.

### 4.5. Preparative HPLC Separation

The preparative HPLC system Nexera was used for the final purification of the fraction obtained from CPC separation that contained E-8-*O*-G. The instrument was composed of a liquid chromatograph 20AP, UV detector SPD-M40, system controller CBM-40, thermostat CTO-40C, autosampler, and fraction collector LH-40 (Shimadzu, Kyoto, Japan). The separation was performed on an RP 18 preparative chromatographic column ReproSil (250 × 20 mm, 5.0 µm). The following gradient elution of acetonitrile (eluent A) and water (eluent B) was used in the flow rate of 14 mL/min: 0 min—30% A, 35min—60% A. Next, the column was rinsed with 95% acetonitrile for the following 13 min. The injection was performed using three CPC fractions containing E-8-*O*-G that were mixed together, evaporated, and re-dissolved in 30% acetonitrile at the concentration of 20 mg/mL. A total of 1 mL of sample was injected into the chromatograph each time. The UV detector was operated at three wavelengths, namely 254, 280, and 278 nm. The fractions were collected manually according to the composition of the chromatogram.

### 4.6. Cell Lines

The SK-N-AS (ATCC^®^ CRL-2137™) neuroblastoma, T98G (ATCC^®^ CRL-1690™) human glioblastoma, and C6 (ATCC^®^ CCL-107™) mouse glioma cell lines were obtained from the American Type Culture Collection (ATTC) (Manassas, VA, USA). Tumor cells were maintained in DMEM/HAM F12 culture medium (Sigma-Aldrich; Saint Louis, MO, USA) supplemented with 10% fetal bovine serum (FBS) (Sigma-Aldrich), and antibiotics: penicillin (100 IU/mL), and streptomycin (100 µg/mL) (Sigma-Aldrich). Mycoplasma-free cultures were kept in a humidified atmosphere of 95% air and 5% CO_2_ at 37 °C.

### 4.7. MTT Cell Viability Assay

The viability of nervous system tumor cells after E-8-*O*-G treatment was experimentally evaluated in the MTT (3-(4,5-dimethylthiazol-2-yl)-2,5-diphenyltetrazolium bromide) assay. The SK-N-AS, T98G, and C6 cancer cells (2 × 10^4^ cells/mL) were plated in 96-well plates (Nunc, Roskilde, Denmark), and then treated with E-8-*O*-G in the concentration range of 5–200 µM for 96 h. Subsequently, the cells were incubated for 3 h with MTT solution (5 mg/mL) (Sigma-Aldrich). During this time, MTT was metabolized by living cells to purple formazan crystals, which were later dissolved in SDS buffer (10% SDS in 0.01 N HCl). The optical density of the resulting product was measured spectrophotometrically at 570 nm using an Infinite M200 Pro microplate reader (Tecan; Männedorf, Switzerland).

### 4.8. CellTiter-Glo^®^ Cell Viability Assay

The viability of nervous system tumor cells after E-8-*O*-G treatment was experimentally assessed in the CellTiter-Glo^®^ assay. CellTiter-Glo^®^ is a luminescent cell viability test based on the quantification of ATP (adenosine triphosphate) release, an indicator of metabolically active cells. SK-N-AS, T98G, and C6 tumor cells at 2 × 10^4^ cells/mL density were plated in 96-well plates and then treated with E-8-*O*-G in the concentration range of 1–150 µM for 48 h. After this time, 100 µL of CTG reagent was added per well, the plate was shaken for 2 min, and incubated for 15 min. Luminescence was measured at 1/minute using the Infinite M200 Pro microplate reader (Tecan).

### 4.9. BrdU ELISA Cell Proliferation Assay

The SK-N-AS, T98G, and C6 tumor cells were placed on 96-well plates (Nunc) at a density of 1 × 10^4^ cells/mL. After 24 h, the cells were treated with 5–200 µM of E-8-*O*-G for 48 h. DNA synthesis in proliferating cells was evaluated by measurement of 5-bromo-2′-deoxyuridine (BrdU) incorporation using a Cell Proliferation ELISA, BrdU kit (Roche; Basel, Switzerland). The absorbance of the final product was measured with the Infinite M200 Pro microplate reader (Tecan) at 450 nm.

### 4.10. Statistical Analysis

The statistical analysis was performed by the one-way analysis of variance (ANOVA) test for multiple comparisons followed by Tukey’s significance test in GraphPad Prism 6 Software. Data are expressed as the mean ± standard deviation (±SD). * *p* < 0.05, ** *p* < 0.01, *** *p* < 0.001.

## 5. Conclusions

The manuscript shows the isolation protocol used for the recovery of emodin 8-*O*-glucoside from the aerial parts of an invasive ruderal plan, namely *Reynoutria japonica*. The species was found to contain a multitude of pharmacologically important polyphenols and anthracene derivatives. The application of centrifugal partition chromatography (CPC) operated in the descending mode using a mixture of petroleum ether: ethyl acetate: methanol: water (4:5:4:5 *v*/*v*/*v*/*v*) and subsequent purification with preparative high-performance liquid chromatography (HPLC) resulted in the isolation of E-8-O-G in high purity and a sufficient quantity for bioactivity testing.

In the conducted studies, E-8-*O*-G inhibited the viability and induced cytotoxicity of neuroblastoma and glioblastoma cancer cells in the concentration range of 1–200 µM. The highest activity of E-8-*O*-G was observed against C6 mouse glioblastoma cell line with IC_50_ = 52.67 µM. The IC_50_ for T98G human glioblastoma cells and SK-N-AS neuroblastoma cells were 61.24 µM and 108.7 µM, respectively.

The confirmed anticancer potential of the isolated emodin glucoside shows a possible application of this invasive plant as a source of pharmacologically potent metabolites and qualify this compound for further pharmacological evaluation. However, more advanced molecular studies are needed to elucidate targets of E-8-*O*-G.

## Figures and Tables

**Table 1:** The proposed identification of the major constituents of *Reynoutria japonica* extract in the HPLC-ESI-QTOF-MS/MS analysis.

No	Compound	Formula	Rt (min)	Pos/Neg (−/+)	Theoretical Mass	Experimental Mass	Error	DBE	MS/MS Fragments	$\mathrm{Amount}\times 10^{6}$
1	Emodin	C_15_H_10_O_5_	24.4	−	269.0455	269.0446	3.51	11	241, 225	9
2	Emodin-6-*O*-glucoside	C_21_H_20_O_10_	21.1	−	431.0984	431.1003	−4.47	12	304, 311, 283, 269	1.6
3	Emodin-8-*O*-glucoside	C_21_H_20_O_10_	21.3	−	431.0984	431.0994	−2.38	12	341, 311, 283, 271	1.4
4	Physcion	C_16_H_12_O_5_	22.4	−	283.0612	283.0626	−4.94	11	112, 248	0.11
5	Quercetin	C_15_H_10_O_7_	22.3	−	301.0354	301.0368	−5.38	11	151, 178, 273, 107	8
6	Rutin	C_27_H_30_O_16_	21.1	−	609.1461	609.1482	−4.58	13	-	0.6
7	Luteolin	C_15_H_10_O_6_	22.7	−	285.0405	285.0419	−5.03	11	241, 268	1.5
8	Luteolin 7-*O*-glucoside	C_21_H_20_O_11_	20.591	−	447.0933	447.0950	−3.83	12	429, 357, 327, 285	1.6
9	Citric acid	C_6_H_8_O_7_	3.672	−	191.0197	191.0205	−4.03	3	111, 87	8
10	Gallic acid	C_7_H_6_O5	16.174	−	169.0140	169.0154	−6.78	5	151, 125, 83	7
11	Tartaric acid	C_4_H_6_O_6_	18.257	−	149.0092	149.0096	−2.92	2	-	0.6
12	Cafraic acid	C_13_H_12_O_9_	18.257		311.0409	311.0426	−5.59	8	-	5
13	Bergapten	C_12_H_8_O_4_	2.006	−	215.0350	215.0331	11.95	9	181, 89	7
14	Malic acid	C_4_H_6_O_5_	2.922	−	133.0142	133.0139	2.59	2	115, 71	1.5
15	Coumarin	C_9_H_6_O_2_	19.085	+	147.0441	147.0440	0.38	7	-	0.4
16	Catechin	C_15_H_14_O_6_	20.418	+	291.0863	291.0868	−1.67	9	-	0.3
17	Procyanidin B1	C_30_H_26_O_12_	19.918	+	579.1497	579.1498	−1.03	18	-	4.5
18	Quinic acid	C_7_H_12_O_6_	2.089	−	191.0561	191.0564	−1.5	2	-	0.5
19	3-*p*-coumaroylquinic acid	C_16_H_18_O_8_	19.174	−	337.0929	337.0933	−1.21	8	163, 191	3
20	Isoquercetin	C_21_H_20_O_12_	21.4	−	463.0882	463.0901	−4.09	12	317, 300, 255	1.6
21	Sacranoside A	C_20_H_32_O_10_	19.8	−	431.1995	431.1950	−6.3	5	387, 179	7
22	Procyanidin B6	C_30_H_26_O_12_	19.9	−	577.1351	577.1374	−3.89	18	426, 289, 194	7
23	Isoschaftoside	C_26_H_28_O_14_	20.6	−	563.1406	563.1436	−5.27	13	521, 483, 303, 263	3

**Table 2:** The composition of biphasic solvent system used for the study.

No	Solvent System	Ratio	References
1	Petroleum ether:ethyl acetate:water	1:5:5	[26]
2	Hexane:ethyl acetate:methanol:water	3:7:5:5	[25]
3	Hexane:ethyl acetate:methanol:water	9:1:5:5	modified
4	Hexane:ethyl acetate:methanol:water	8:2:6:4	modified
5	Hexane:ethyl acetate:methanol:water	8:2:9:1	modified
6	Ethyl acetate:methanol:water	70:1:70	[26]
7	Petroleum ether:ethyl acetate:methanol:water	4:5:4:5	[27]

## Data Availability

Not applicable.

## Supplementary Materials

The following supporting information can be downloaded at: https://www.mdpi.com/article/10.3390/molecules28217366/s1. Table S1. MS/MS spectra of the identified compounds; Table S2. The partition coefficients of main compounds of Reynoutria japonica extract.
