# Supplementary material for: Emodin-8-O-Glucoside—Isolation and the Screening of the Anticancer Potential against the Nervous System Tumors
Source: Molecules. 2023 Oct 31;28(21):7366. doi: 10.3390/molecules28217366 (PMC10650745; doi:10.3390/molecules28217366)
Supplement: Supplementary file 1 [file molecules-28-07366-s001.zip › molecules-2643727-supplementary.pdf]

SUPPLEMENTARY FILE

Table S1. MS/MS spectra of the identified compounds.

<table>
<tr><td>Emodin</td><td>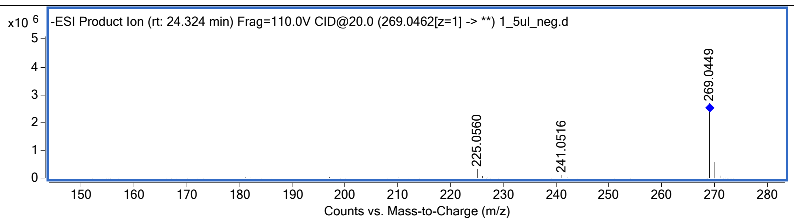
</td></tr>
<tr><td>Emodin-6-O-glucoside</td><td>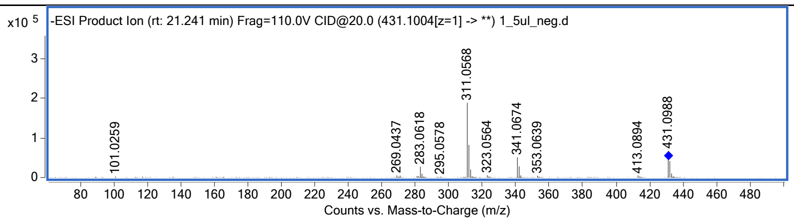
</td></tr>
<tr><td>Emodin 8-O-glucoside</td><td>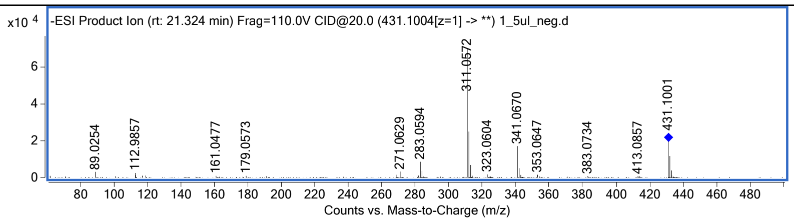
</td></tr>
<tr><td>Isoquercetine</td><td>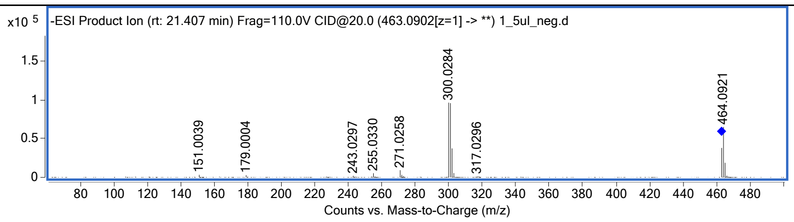
</td></tr>
<tr><td>Luteolin glucoside</td><td>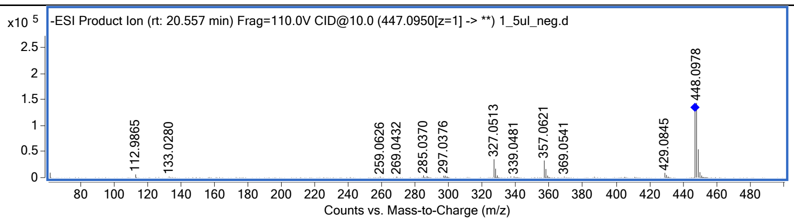
</td></tr>
</table>

Sacranoside A	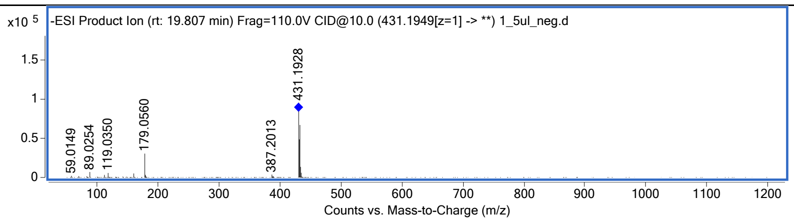
Procyanidin B6	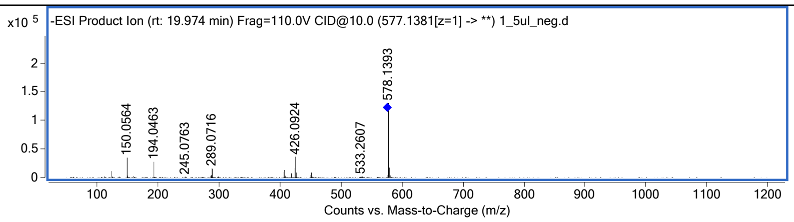
Quercitin	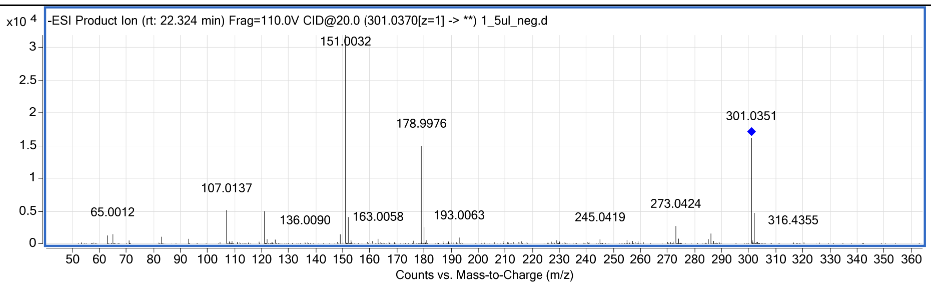
Luteolin	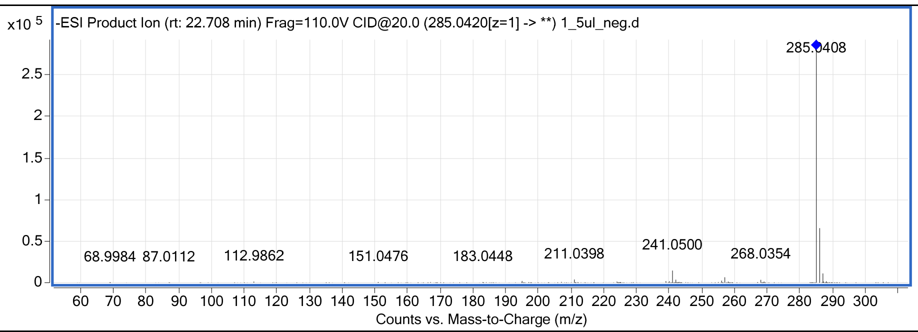
Citric acid	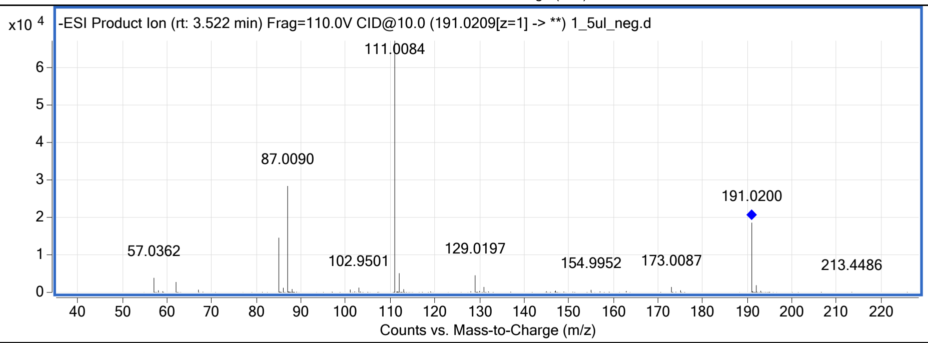

Gallic acid

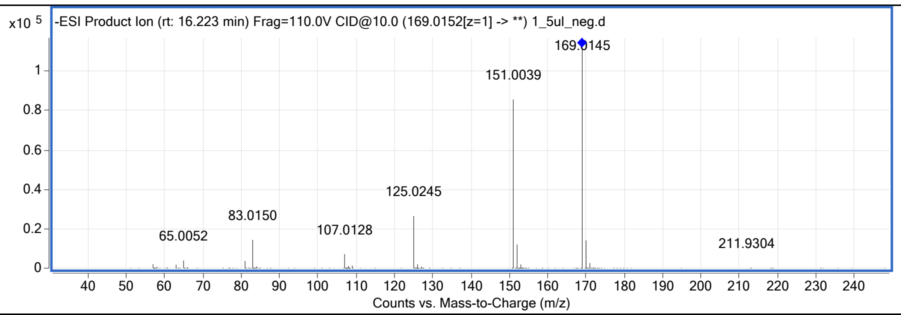

Bergapten

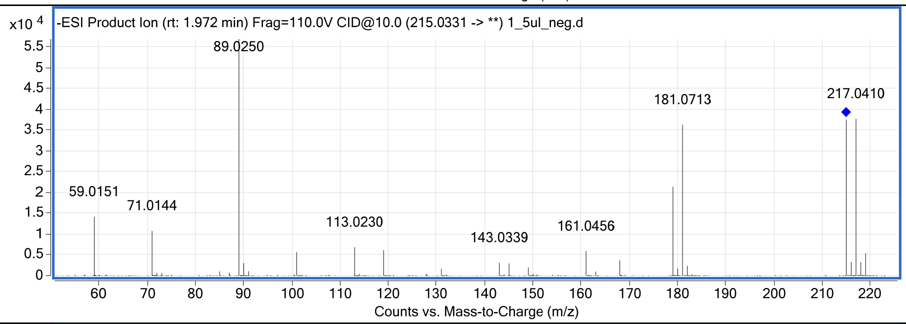

Malic acid

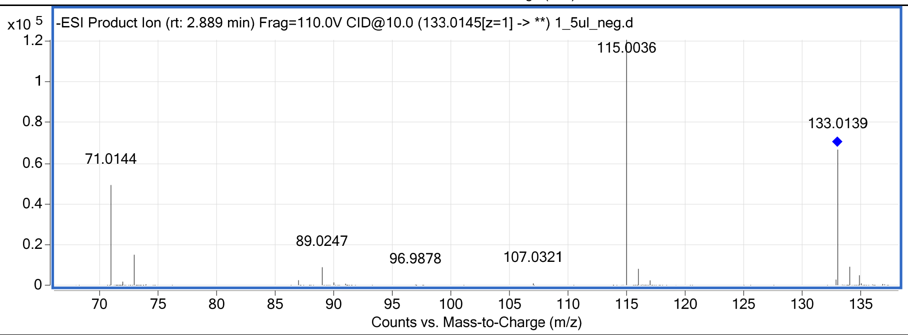

Citric acid

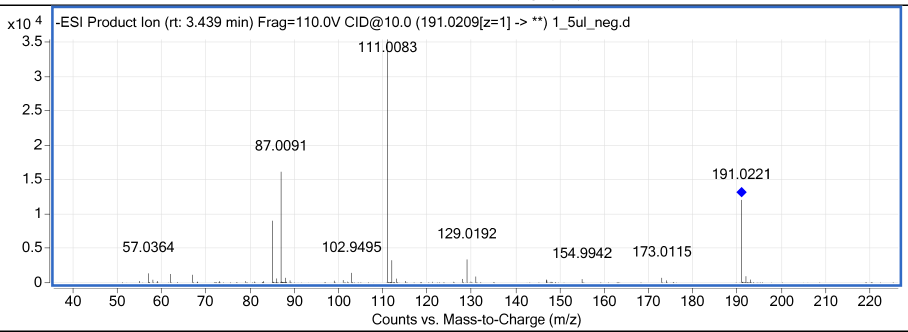

3-p-coumaroylquinic acid

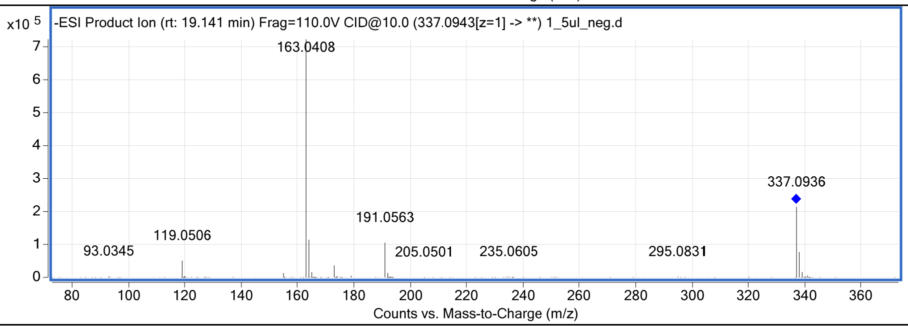

Isochaftoside	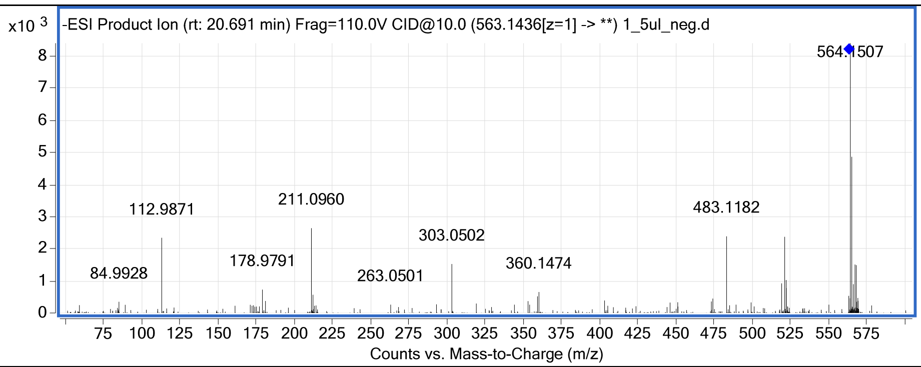
Isoquercitin	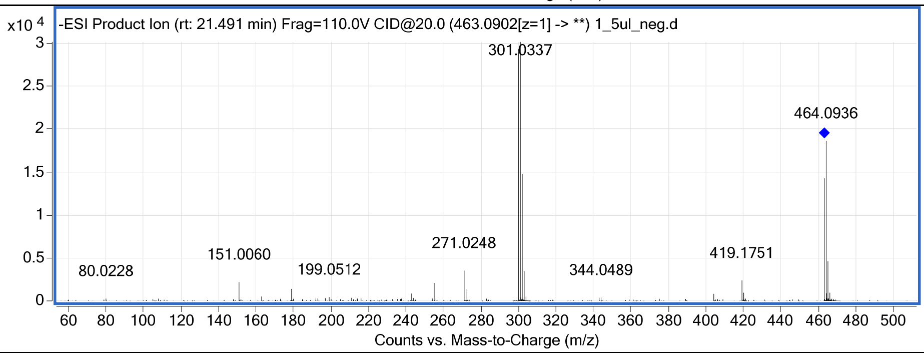

Table S2. The partition coefficients of main compounds of *Reynoutria japonica* extract.

Solvent system	RT	Upper	Lower	K
1	18.6-18.8	58097239.82	1340267.37	43.3475
	2.8-2.9	2099046.97	10450229.62	0.200861325
	14.7	518177.37	518177.37	1
	13.4-13.7	3059086.66	525192.05	5.824701002
	2-2.3	18210901.09	6482046.5	2.809436972
2	14	3104945.16	8937374.49	0.34741133
	19.1-19.2	6220079.69	3102510.49	2.004853718
	13.4	1372222.59	1953007.11	0.702620376
	31.3	20910055.67	25363253.51	0.824423241
	24.7	802523.21	14454896.31	0.055519126
	23.8-23.7	8085243.89	981185.87	8.240277543
3	18.3-18.5	1125871.95	1438062.8	0.782908751
	18.6-19.1	11758022.84	4266784.77	2.755710324
	30	2002144.87	3214375.3	0.622872155
	31.4-31.6	18974832.23	6890689.74	2.753691277
4	18.5	162945.97	162404.71	1.003332785
	18.7-19	1102254.22	711543.22	1.549103679
	29.6	332057.43	131645.01	2.522370047
	31.4-31.2	80732.12	237487.03	0.33994328
5	18.4	118222.13	192285.57	0.614825803
	18.7-19	1035649.51	605339.36	1.710857708
	21.8-21.1	9957.76	41411.08	0.240461249
	22.7	64475.67	33757.41	1.909970878

6	2	150954.64	240360.7	0.628033784
	3.3-3.2	173928.03	16286.24	10.67944658
	13.5-13.4	300317.96	300317.96	1
	14	919370.9	210446.15	4.368675312
	15.6-15.3	300317.96	6278.24	47.83473712
	19	1462667.17	23092.13	63.34050475
	19.9-20.2	128859.81	29786.92	4.326053516
7	18.8-19.1	1416808.58	452956.96	3.127909945
	19.8-19.9	98109.77	56558.8	1.734650841
	22.2-22.7	30719.87	190708.7	0.161082688
	15.4-15.2	141592.91	64657.68	2.189885409

(RT – retention time, Upper – peak area in the upper phase, Lower – peak area in the lower phase, K – the calculated partition coefficient value that was obtained by dividing Upper by Lower values)
